# eDNA Metabarcoding Reveals the Species–Area Relationship of Amphibians on the Zhoushan Archipelago

**DOI:** 10.3390/ani14111519

**Published:** 2024-05-21

**Authors:** Wenhao Li, Xianglei Hou, Yunlong Zhu, Jiacong Du, Chunxia Xu, Jingyuan Yang, Yiming Li

**Affiliations:** 1 Key Laboratory of Animal Ecology and Conservation Biology, Institute of Zoology, Chinese Academy of Sciences, Beijing 100101, China; 2 University of Chinese Academy of Sciences, Beijing 100049, China; 3 State Key Laboratory of Integrated Management of Pest Insects and Rodents, Institute of Zoology, Chinese Academy of Sciences, Beijing 100101, China; 4 School of Life Sciences, Hebei University, Baoding 071002, China; 5 Shengnongjia National Park Administration, Huibei Provincial Key Laboratory on Conservation Biology of the Shennongjia Golden Snub-Nosed Monkey, Shennongjia 442421, China

**Keywords:** amphibians, biodiversity, eDNA, metabarcoding, species richness, island biogeography, species–area relationship

## Abstract

**Simple Summary:**

This study investigates the species–area relationship of amphibians on the Zhoushan Archipelago using eDNA metabarcoding. By analyzing amphibian species diversity on the Zhoushan Archipelago, the eDNA metabarcoding detected eight amphibian species on the islands and nine species in the mainland areas. Findings reveal that the amphibian species diversity is positively related to the island area. In comparison with the traditional line transect method, eDNA metabarcoding detected more amphibian species. eDNA showcases enormous potential in detecting amphibian species.

**Abstract:**

The species–area relationship is important for understanding species diversity patterns at spatial scales, but few studies have examined the relationship using environmental DNA (eDNA) techniques. We investigated amphibian diversity on 21 islands of the Zhoushan Archipelago and nearby mainland areas in China using the combination of eDNA metabarcoding and the traditional line transect method (TLTM) and identified the species–area relationship for amphibians on the islands. The mean detection probability of eDNA is 0.54, while the mean detection probability of TLTM is 0.24. The eDNA metabarcoding detected eight amphibian species on the islands and nine species in the mainland areas, compared with seven species on the islands and nine species in the mainland areas that were identified by TLTM. Amphibian richness on the islands increased with island area and habitat diversity. The species–area relationship for amphibians in the archipelago was formulated as the power function (S = 0.47A^0.21^) or exponential function (S = 2.59 + 2.41 (logA)). Our results suggested that eDNA metabarcoding is more sensitive for the detection of amphibian species. The combined use of eDNA metabarcoding and the traditional line transect method may optimize the survey results for amphibians.

## 1. Introduction

The species–area relationship is one of the basic laws of ecology [1]. This relationship is commonly employed to examine the correlation between spatial scale and species diversity and plays an important role in understanding the biogeography, heterogeneous population biology, and evolutionary ecology of species [2]. In addition, conservation biologists use the species–area relationship to predict the decline in species diversity when habitat loss occurs and to formulate conservation strategies for biodiversity in nature reserves and fragmented habitats.

Tjrve [3] summarized the species–area relationship into six convex functions and eight sigmoid functions and suggested that power and exponential functions were the most common. Some scholars also found that power functions fit well [4]. In recent years, some researchers have stated that the above function is not suitable for small islands because when the island area is less than a certain threshold, species diversity may not be related to the area but determined by random factors, which is referred to as the “small-island effect”. Lomolino used a function with a breakpoint to fit the species–area relationship [5].

The relationship between island area and species diversity is complex [6]. The area of an island can affect species diversity in two ways [7]: (1) for individuals with strong dispersal ability, a large island provides a large target, and, thus, the colonization rate is high; and (2) when the population of a species on a large island is large, the extinction probability of that species is low. Other factors related to island area, such as habitat diversity, can also indirectly affect species diversity. Large islands usually have higher habitat diversity, which increases species diversity, especially for habitat specialists [8]. The degree of isolation of an island is usually related to the distance from the island to the mainland and can also indirectly affect the species diversity on the island, especially for strongly migratory species. An efficient biodiversity survey method can help us understand the relationship between island area and species diversity.

Biodiversity surveys may essentially monitor changes in biodiversity over spatiotemporal scales. The information obtained from these surveys can provide a scientific basis for the development of biodiversity assessments and future biodiversity monitoring strategies. The traditional line transect method (TLTM) is currently being used as a conventional technique for biodiversity assessment and long-term monitoring in terrestrial and aquatic ecosystems. Compared with methods using infrared cameras and drift fences, the TLTM has a lower environmental impact and requires less time investment [9]. Additionally, traditional biodiversity monitoring methods based on acoustic or visual sampling are often limited in what they can detect [10,11,12]. Researchers need a method that can detect uncommon, elusive, invasive, and endangered species. This method should offer high accuracy at low cost and no environmental impact.

Environmental DNA (eDNA) is the DNA released into the environment by organisms through feces, fur, urine, skin, gametes, and other means [13]. This type of DNA can be extracted from environmental samples such as soil, water, and feces without the need to isolate the target organism [13,14]. The eDNA method relies on the fact that all species leave DNA traces in their environment, and these DNA traces can be collected. This method has been widely used in microbiology for many years [15]. However, it was recently introduced and gained popularity in amphibian and fish assessment [16,17,18,19]. The application of metabarcoding in an ecosystem is useful not only for describing the community and biodiversity but also for detecting the interaction between populations at a large spatial scale. However, this technique may be limited by false readings caused by contamination or other errors [20,21,22,23,24,25]. In other words, compared with those of traditional sampling methods, metabarcoding has improved detection speed, accuracy, and identification ability and has a reduced cost. However, at present, metabarcoding needs to be standardized and unified, combining taxonomy and molecular methods for more comprehensive ecological research [26].

Amphibians are among the most vulnerable taxa in the world [27]. Identifying the species–area relationship for amphibians on islands will help reveal the impacts of long-term island habitation on amphibian biodiversity and provide a basis for species protection strategies [28]. In this study, we investigated amphibian diversity on 21 islands of the Zhoushan Archipelago in East China Sea and three nearby regions in mainland China (in Zhejiang Province) using the traditional line transect method (TLTM) and eDNA metabarcoding. We examined the effectiveness of metabarcoding for the monitoring of amphibian diversity across all island and mainland sample sites and compared differences in the effectiveness between the two methods. Finally, we identified the species–area relationship of amphibians on the islands.

## 2. Materials and Methods

### 2.1. Study Area and Sample Location

**Figure 1:**
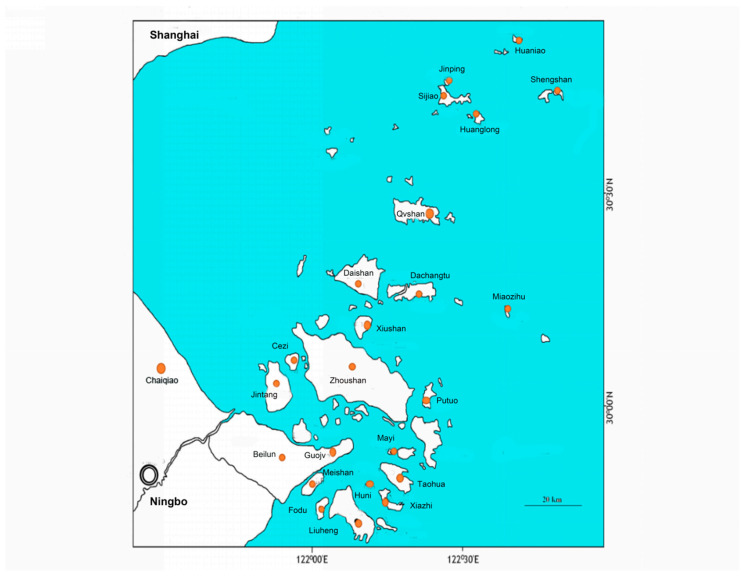
Study area in the Zhoushan Archipelago and the nearby mainland.

The Zhoushan Archipelago is located on the eastern coast of Zhejiang Province and is China’s largest archipelago, consisting of 1339 islands with an area > 500 m^2^. Zhoushan Island is the largest (467.8 km^2^) amongst these islands. Beilun is located at a latitude of 29°41′–30°1′ N and longitude of 121°39′–122°10′ E. We chose 21 islands that are easily accessible by boat. Both the traditional line transect method and eDNA metabarcoding technology were employed to sample from the 21 selected islands within the Zhoushan Archipelago and three regions within the Beilun mainland for anurans. There were 29 sampling sites for each mainland region, 14 sampling sites for each large island, 11 sampling sites for each medium-sized island, and 5 sampling sites for each small island. A total of 288 sites were sampled (Figure 1, Table 1 and Table S1). Both traditional and metabarcoding sampling were conducted in the same year (2018). To prevent possible bias arising from variable sampling efficiency across sites, anuran species abundance data were converted into occurrence data for each site.

### 2.2. Traditional Methods

From 12 July to 15 September 2018, we investigated the richness and abundance of amphibians in water bodies by traditional line sampling methods. This method is based on visual and acoustic sampling [28,29,30,31,32]. After eDNA sampling, we searched for amphibians along the transect every night from 19:30 to 22:30. Observers walked slowly and carefully along each transect (1–2 km/h) with a flashlight (12-volt DC lamp) (Qingtianzhu 201, Northwestern Light Industry Company, Xian, China), counting all the amphibian postmetamorphic individuals and tadpoles. Each line transect was 100 m long and 2 m wide. We also carefully listened to calls from amphibians along the transects. To avoid counting the same individual more than once, we recorded only the individuals that we visually observed when we both saw individuals of a species and heard calls from that species along a transect. We sampled frogs or tadpoles using the traditional line transect method and eDNA sampling.

### 2.3. Reference DNA Database

We have defined a list of 9 amphibians that have been detected in our aquatic eDNA samples based on the literature reports on species diversity and known species ranges, known to be distributed in Zhoushan Island and the Beilun continent. We have constructed a reference database of 12S rRNA mitochondrial sequences as fully as possible to ensure appropriate classification and allocation of sequences recovered from environmental samples. Following the extraction of total genomic DNA from toe clip tissues (1–3 individuals per species) for 9 amphibian species (Table 2), we amplified the 12S rRNA mitochondrial gene in a dedicated room. We sequenced the PCR products using Sanger sequencing (MyGenotics, Beijing, China). All PCR primers and cycling conditions followed those of Li et al. (2021)’s study [16].

We supplemented the local sequence reference database with sequences recovered from the EMBL database (version 143). We downloaded all vertebrate sequences from the EMBL database. We extracted relevant fragments of the 12S sequence from our local reference database and EMBL database using ecoPCR 0.5.0 [33] and OBITools 1.1.22 [34]. Considering the available organization and EMBL materials, the final 12S metabolic barcode reference database should be as complete as possible.

### 2.4. eDNA Metabarcoding Sampling

We sampled the water in the water body between 18:30 and 19:30 every night for eDNA analysis. For each sample water body, we selected 5 sampling points along the riverbank and collected 400 mL of surface water at each sampling point using sterile bottles. For each water body, we repeated the experiment three times. Then, we mixed one replicate of each of the 5 samples into a bottle to obtain three bottles, each containing 2000 mL of water.

We use a 1.5 µm glass fiber filter to filter each water sample until the filter was clogged. In order to control for possible equipment pollution or cross contamination between water bodies, a negative control was established by filtering 1000 mL of ultrapure water (one filter) (Lan Hai Co., Ltd., Guangzhou, China). We stored the filter in a 1.5 mL spiral cap microcentrifuge tube in ethanol and placed it on ice. Before extracting DNA, all test tubes were stored in the laboratory at a temperature of −20 °C. Before sampling each water body, we used 10% commercial bleach (approximately 0.5% hypochlorous acid) to remove any DNA attached to the bottles and filtration equipment, and then cleaned the bottles with distilled water that did not contain DNA. The samples of each water body were collected and filtered by individuals wearing disposable powder-free latex gloves.

We extracted the total genomic DNA from the filters of each water sample using a General AllGen Kit (Beijing CoWinBiotech Co., Ltd., Beijing, China) [35], which generated 100 µL of DNA extract. We amplified DNA from a short fragment of the 12S rRNA mitochondrial gene in a mixture that included 2 µL of DNA extract as a template, 1 U of Start Taq DNA Polymerase (General AllGen), each dNTP(General AllGen) at 0.2 mM, the batra_F (5′-ACACCGCCCGTCACCCT-3′) and batra_R (5′-GTAYACTTATGTTACTT-3′) primers at 0.2 µM, and the human-specific blocking primer batra_blk (5′-TCACCCTCCTCAAGTATACTTCAAAGGCA-SPC3I-3′) at 4 µM [36]. The “batra” primers were used for amplification because they showed high taxonomic discrimination [36]. For all studied species, we tested the “batra” primers on DNA isolated from tissues, and for NGS sequencing, the species in this study were correctly identified. The primers (batra_F and batra_R) were 5′ labeled with a unique six-nucleotide tag (with at least three differences between tags) to differentiate all the samples. Sequences were assigned to the individual samples during the sequence filtering process by the primer tags. The initial denaturation step was performed at 94 °C for 5 min, followed by 35 cycles of 30 s at 94 °C, 30 s at 58 °C, and 30 s at 72 °C. The final elongation step was performed at 72 °C for 10 min. The PCR was run for 9 replicates per sample. Additional negative controls were generated using ddH_2_O (instead of the filters) during DNA extraction. Following the methods of Dejean [37], we used DNA extracted from *Lithobates catesbeianus* as a positive control to verify whether the amplification of DNA from the target species in the eDNA samples failed due to PCR inhibition. We ran parallel PCR assays of the negative (including negative field controls and PCR processing controls) and positive controls. DNA extraction and PCR amplification were performed in isolated rooms.

Before preparing the library, we used gel electrophoresis to check the quality of each PCR product according to the fluorescence signal. All negative controls (negative field control and PCR treatment control) showed a lack of PCR products after gel electrophoresis. We collected each PCR product in an equal volume to construct a library with an expected sequencing depth of 300,000 reads per sample. We used a DNA sample preparation kit (MyGenotics, Beijing, China) to prepare a library, including end repair and linker linkage. We established 9 polymerase chain reaction product libraries from water samples, each library containing 32 samples (a total of 288 samples), two libraries for negative controls (one for polymerase chain reaction control and one for on-site control), and one library for positive controls. According to the manufacturer’s plan, the DNA of the library was further purified before sequencing using Solid Phase Reversible Immobilization (SPRI) beads (Beckman Colter, Brea, CA, USA). The enriched library was sequenced on an Illumina HiSeq X Ten sequencer, with a paired end reading of 150 bp.

We used the functions implemented in the OBITools package to filter and annotate eDNA sequence reads [34]. We assembled direct and reverse chains to construct a shared sequence, and then used the Illumina pairing end program to remove low-quality sequences from the shared sequence (FASTQ average quality score < 35). We identified each sequence record based on its molecular label (no errors allowed) and used the “ngsfilter” function to assign primers (each primer allows 2 bp errors) to PCR products using the corresponding samples. Unaligned sequences were removed from the dataset using the “obigrep” function. For each library, we used the “obiuniq” function to cluster strictly identical sequences into operational classification units (OTUs) to preserve the information in their read counts. Using the “obigrep” function, we removed sequences that were too long (>130 bp) or too short (<30 bp), removed OTUs with read counts ≤ 10, and only retained OTUs with total read counts > 10 for denoising analysis. We used the “obiclean” function [34] to label the sequences in each library as “head”, “singleton”, or “internal” (likely due to amplification/sequencing errors and chimeric sequences). Then, we removed the “singleton” and “internal” sequences from each library, leaving only the “head” sequence for classification allocation (option—r 0.05-H) [38]. We downloaded gene sequence data from the EMBL database (version 124) of the National Center for Biotechnology Information (NCBI); these data include information on different taxonomic groups (vertebrates, mammals, prokaryotes, fungi, invertebrates, and plants) and environmental samples. We constructed an NCBI database of 12S rRNA mitochondrial sequences using ecoPCR based on downloaded data [33]. We classified the sequences of samples and negative and positive controls based on the NCBI reference database and the local amphibian reference database using ecotag tools. We assigned sequences to database sequences based on a 95% similarity threshold. We carefully verified target species detection to avoid false positives. We considered the ability of 12S rRNA to differentiate local species. We also tested whether the DNA traces of the target species detected at the survey site were consistent with the species investigated by traditional methods in the study environment.

### 2.5. Statistical Analyses

We compared the species list obtained from eDNA sampling with the species list obtained from traditional sampling. For each sampling point, we calculated the species occurrence rate detected by each method; specifically, we divided the number of sampling points detected by a method by the total number of sampling points.

For the whole sampling area, we used the dilution curve to fit the relationship between the expected species richness of the eDNA method and traditional line transect method and the expected sampling workload. We compared the dilution curves between the traditional line transect method and metabarcoding method for each sample. We also fitted the sampling workload required by each of the two methods to observe all species in all studied waterbodies. In the “vegan” package of R language version 3.3.2, the “rich” and “rarc” functions were used for 2000 random-simulation dilution analyses. All statistical analyses were performed using R 3.3.2 (R 2015). The significance level was set at *p* < 0.05.

The two common species–area relationship forms are as follows: power function,

log S = c + z(log A) or S = cA^z^ (1)

and exponential function,

S = k0 + k1(log A)(2)

In these formulas, S is the number of species, A is the area of the island, and C, Z, k0, and k1 are constants. We fit the frog data from the Zhoushan Islands with Formulas (1) and (2) to determine the most suitable form of the species–area relationship.

To determine the factors that affect the species diversity of the island, we used the single-factor correlation and stepwise regression method to test the impact of the island area (log conversion), the island altitude (log conversion), the distance from the island to the mainland (log conversion), and the distance from the island to the nearest large island (log conversion) to determine the main factors that affect the diversity of amphibians.

We used simple linear regression with a breakpoint transformation to estimate the upper limit of the small-island effect (SIE) along with r^2^ values. The breakpoint, or piecewise regression model with two pieces, used is described below:

Y = b0 + b1[(log(A)−T) × (log(A) ≥ T)](3)

where Y is the species richness (S) or log 10 (S) for the model, A is the island area (here, in km^2^), T is the upper limit of the SIE, and (log 10 (A) ≥ T) is a variable that returns the logical value of 0 or 1.

On islands smaller than T, the independent variable is equal to 0, and the species richness is estimated to be a constant (b0), independent of the island area. On larger islands with log 10 (A) exceeding T, the independent variable is equal to the difference between log 10 (A) and breakpoint T. The data for each archipelago are not divided into large and small islands, but they are included in a linear regression model for each archipelago or dataset. The sample size is equal to the total number of islands, and the estimates of intercept, slope, and coefficient of determination are applicable to the entire dataset, assuming the assumption of a relatively robust general linear model. The key difference between this model and more traditional models is the breakpoint transformation, which basically reduces the x values of all small islands to 0 and reduces the upper limit T of SIE for all larger islands.

For each amphibian species at each sample site, if detected by eDNA or the TLTM, the sample site was considered to be occupied. We used a maximal r^2^ method to estimate the upper limit of the SIE. We performed 253 regressions for each model and dataset (Table S3), increasing the trial breakpoint by 0.01 each iteration through the range of T = 0.15 (the area logarithm of the smallest island) to 2.67 (the area logarithm of the largest island). The amphibian data were regressed by Formula (3) to obtain the corresponding r^2^ value. When the r^2^ value is at a maximum, the corresponding T value is the threshold of the “island effect”. We used the logarithm of the number of species and the number of species to perform this calculation twice to determine whether there was an “island effect” in the two cases and obtained the corresponding threshold.

## 3. Results

### 3.1. Detection Efficiency of eDNA and the Traditional Line Transect Method

**Figure 2:**
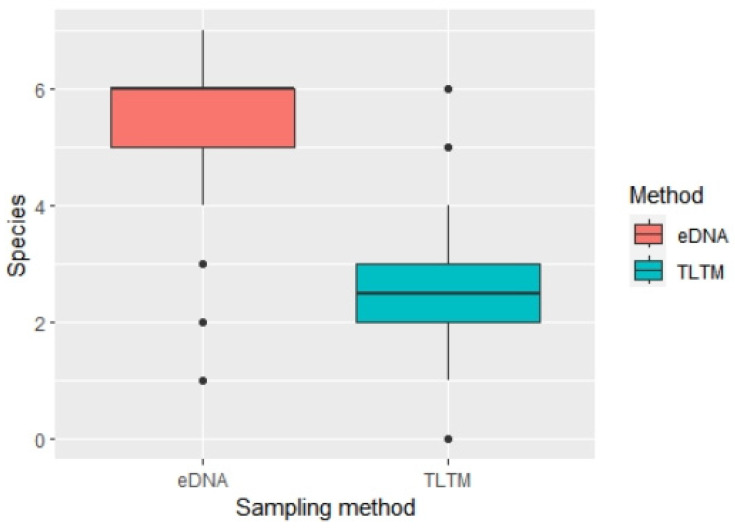
Species richness per site as detected with the traditional and eDNA metabarcoding methods.

When using eDNA, a total of nine amphibian species were detected (Table 2). Among the sampling sites, the average species richness of amphibians for each sampling site was 5.47 ± 1.21 (range 1–7 species). When using the traditional line transect method, a total of nine amphibian species were detected (Table 2). Among the sample sites, the minimum was 0 species, the maximum was 6 species, and the mean was 2.55 ± 1.35 species (Figure 2, Table S2). Species occurrence rates among the sample sites when using eDNA resulted in a minimum of 0, a maximum of 1, and a mean occurrence of 0.54 ± 0.46. When using the traditional line transect method, among the sampling sites, the minimum was 0, the maximum was 0.86, and the mean occurrence was 0.24 ± 0.26. All amphibians’ detection probability by eDNA is higher than that by the TLTM (Table 2 and Table S2).

**Figure 3:**
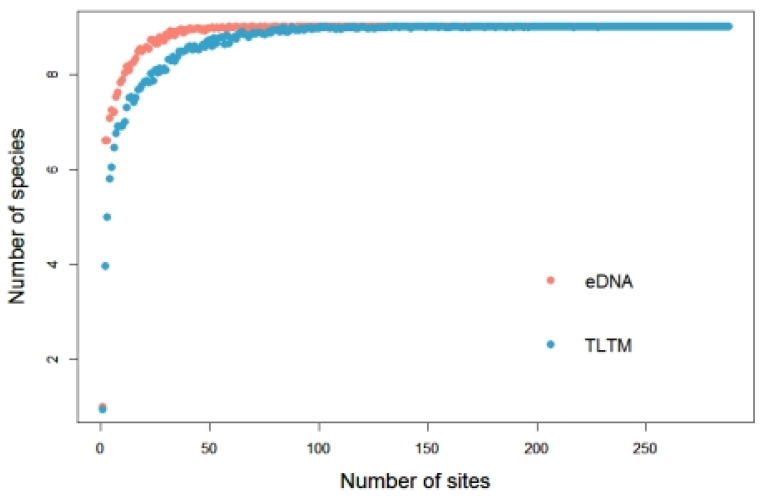
Rarefaction curves of species richness based on the number of waterbodies sampled using the two methods.

The rarefaction curve shows that the sampling efforts required by the two methods to detect species richness in the whole sampling area are different (Figure 3). The waterbodies needed to be sampled only 20–30 times for metabarcoding analysis to obtain a good estimate for all nine amphibian species, while the waterbodies needed to be sampled 80–90 times for the traditional line transect method analysis to obtain a good estimate for all nine amphibian species.

### 3.2. Species–Area Relationship of Amphibians

**Figure 4:**
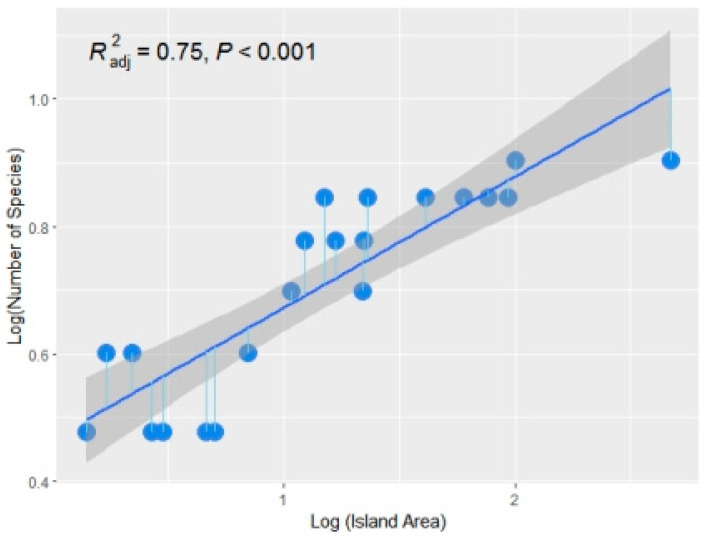
The relationship between amphibian species and island area fitted by Formula (1) (R^2^ = 0.75, *p* < 0.0001). X-axis of blue dots means log(island area), Y-axis of blue dots means number of amphibian species.

**Figure 5:**
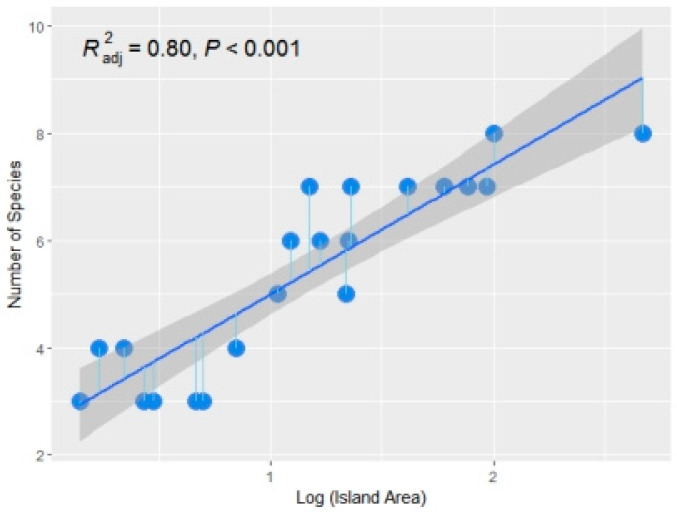
The relationship between amphibian species and area fitted by Formula (2) (R^2^ = 0.80, *p* < 0.0001). X-axis of blue dots means log(island area), Y-axis of blue dots means number of amphibian species.

The relationship between amphibian species and island area fitted by Formula (1) is shown in Figure 4. The regression equation obtained by the least square method is S = 0.47A^0.21^ (R^2^ = 0.75, *p* < 0.0001). The relationship between amphibian species and island area fitted by Formula (2) is shown in Figure 5, and the regression equation is S = 2.59 + 2.41 (logA) (R^2^ = 0.80, *p* < 0.0001).

### 3.3. Factors Affecting the Amphibian Species Diversity of Islands

Table 3 shows that the amphibian species diversity is related to the island area and altitude in the single-factor correlation analysis using the species number logarithm. However, the stepwise regression analysis results show that the main factor affecting the amphibian species diversity is only the island area (R^2^ = 0.76, *p* < 0.001). When using the species number for the single-factor correlation analysis, the amphibian species diversity was related to the island area and altitude. However, when using the stepwise regression analysis, the main factor affecting the amphibian species diversity was only the island area (R^2^ = 0.80, *p* < 0.001).

### 3.4. “Island Effect” on the Amphibian Species–Area Relationship

When performing calculations with the logarithm of the species number, the threshold (T) of the “island effect” was 0.44, and the area corresponding to the T value was 2.7296 km^2^. The regression equation was log S = 0.53 + 0.22 [(log(A) − 0.44) × (log(A) ≥ 0.44)] (R^2^= 0.76, *p* < 0.001).

When fitting with the number of species, the threshold (T) of the “island effect” was 0.48, and the area corresponding to the T value was 2.992 km^2^. The regression equation was S = 3.50 + 2.62 [(log(A) − 0.48) × (log(A) ≥ 0.48)] (R^2^ = 0.80, *p* < 0.001).

## 4. Discussion

Environmental DNA is a promising method for assessing anuran biodiversity in islands and nearby continental areas. The species richness values estimated by the two methods are significantly correlated (r = 0.47, *p* < 0.001). There is also a correlation between the species occurrence rates estimated by the two methods (r = 0.81, *p* < 0.001).

Our research findings confirm that eDNA significantly enhances biodiversity surveys and can be used to overcome many challenges associated with traditional monitoring methods [39]. Due to strict quality control and the careful evaluation of positive testing, eDNA surveys have great potential in detecting low-population-density species and are more capable of detecting hidden species than other methods. Environmental DNA’s detection probabilities for *Sylvirana latouchii*, *Hoplobatrachus rugulosus*, and *Microhyla ornate* are all higher than detection probability of TLTM (Table 2), but it not very significant (*p* ranged from 0.16 to 0.39). This may be because these three species’ detection probability is low (<0.10) in this study (Table 2). Environmental DNA analysis can serve as a rapid assessment method for biodiversity in new tropical regions, and eDNA should be used as a supplementary tool in addition to traditional methods. According to the incidence rate of surveyed species, the number of species detected from eDNA samples is higher than that detected by traditional methods. The proportion of species detected by eDNA is higher than that detected by traditional methods (Table 2 and Table S2).

Like any other survey method, strict quality control of environmental DNA data is crucial to avoid false reporting of missing species (false positives) or undetected species (false negatives) (Table S2). A complete reference database is crucial for avoiding misjudgments, especially in environments with high biodiversity [40].

The Z value of amphibians obtained by logarithmic formula fitting was 0.2063, which is within the general range of the species–area relationship equation (0.20–0.40) [41]. According to the fitting results of the species–area relationship of amphibians, the two formulas are well fitted, but Formula (2) is slightly better than Formula (1), which may be related to the generally small area of the Zhoushan Islands. Some studies [6] have shown that Formula (1) is more suitable for medium-to-large sampling areas, while Formula (2) is more suitable for small sampling areas. Among the Zhoushan Islands, Zhoushan Island has the largest area, which is only 467.8 km^2^, while other islands have areas ≤ 100.0 km^2^. The smallest island in this study, Huni Island, is only 1.4 km^2^, which is a small-to-medium area. This may be the main reason why the fit of Formula (2) was slightly better than that of Formula (1). The amphibian species–area relationship fitted by the two formulas was relatively good. This is mainly because the main factor affecting the diversity of amphibian species is the island area.

Formed in the late Pleistocene, Zhoushan Archipelago was originally a part of Chinese mainland and an extension of Tiantai Mountain in Zhejiang Province. About 9000 years ago, due to rising sea levels and climate change, the archipelago separated from the mainland. The sea level reached its current level approximately 7000 years ago [28]. The main factor affecting the species diversity of amphibians in the Zhoushan Islands is area (*p* < 0.001) (Table 3). Amphibians cannot migrate between islands, which greatly affects their extinction rate [42,43,44,45,46]. Small islands have low species diversity, which is also a core feature of the species–area relationship. Habitat diversity is also one of the influencing factors, although its impact is weaker than that of area. The correlation between habitat diversity and island area was very high. The correlation coefficient between island area and altitude was r = 0.6747 (*p* < 0.0001). During the stepwise regression, altitude was excluded from the equation because island area was included in the equation. According to the results of single-factor analysis, habitat diversity may have an impact on the species diversity of amphibians, but its impact is weaker than that of island area. The results of single-factor analysis show that the distance to the nearest large island has no significant impact on the species diversity of amphibians. Amphibians can migrate between islands [47]. But in this study, amphibians cannot migrate between islands easily [28], and the distance between islands has no impact on their species diversity. In the logarithmic relationship of amphibian species, the fitting degree of Formula (3) was only approximately 1% higher than that of Formula (2), and in the semilogarithmic relationship, the fitting degree of Formula (3) was only approximately 1% higher than that of Formula (2). Thus, the “island effect” was not obvious. The reason may be that the number of smaller islands in our analysis was small. We speculate that the “small-island effect” may exist in amphibians in the Zhoushan Islands, and increasing the number of investigated islands may make the “small-island effect” more obvious.

## 5. Conclusions

Our research findings support the potential application of eDNA in species diversity surveys in island areas. For all amphibian species in the Zhoushan Archipelago, the eDNA method has higher detection probability than the TLTM. eDNA barcoding reveals the amphibian species–area relationship in the Zhoushan Archipelago well. The diversity of amphibians on Zhoushan Island and Beilun Continent is relatively high, while the diversity of amphibians on small islands is relatively low. However, the exact distribution and quantity of these amphibians are still unknown. In future work, eDNA and other survey methods should be applied to describe the geographical range, population fluctuations, and conservation status of species.

## Figures and Tables

**Table 1:** Related natural factors of the Zhoushan Archipelago. DTM = distance to mainland, DTLI = distance to large island.

Site	Area	DTM	DTLI	Altitude	Number of Sampling Sites
Huni	1.4	8.9	2.8	88	5
Jinpin	1.7	56	0.4	132	5
Mayi	2.2	11.5	11.5	157	5
Dongji	2.7	100	100	200	5
Huaniao	3	75.5	15	236.9	5
Shengshan	4.6	90	8.8	213	5
Huanglong	5	68	3.3	223	5
Fodu	7	7	2.3	183	5
Xiaochangtu	10.8	37.6	2.8	299	11
Putuo	12.3	23.8	2.5	291	11
Cezi	14.9	16.5	2.4	275	11
Xiazhi	16.7	13.2	2	207	11
Meishan	21.9	0.5	0.5	148	11
Sijiao	22.3	54.5	45.3	217.8	11
Xiushan	23	26.8	2.3	207	11
Taohua	41	8.8	8.8	539	14
Qushan	59.9	58.5	11.8	250	14
Jintang	76.4	3.6	3.6	455.9	14
Liuheng	92.8	7	7	299	14
Daishan	100	37	12	175	14
Xiepu	195	0	84.7	69.9	29
Chaiqiao	284	0	39.8	502.9	29
Guoju	359	0	36.5	309	29
Zhoushan	468.7	9	9	503	14

**Table 2:** Detection probability with the traditional and eDNA metabarcoding methods. Peni = *Pelophylax nigromaculatus*, Femu = *Fejervarya multistriata*, Buga =*Bufo gargarizans*, Mior= *Microhyla ornate*, Raca = *Rana catesbeiana*, Razh = *Rana zhenhaiensis*, Hych = *Hyla chinensis*, Horu = *Hoplobatrachus rugulosus*, and Syla = *Sylvirana latouchii*.

Species	eDNA	TLTM	*p*
Buga	0.96	0.42	<0.001
Femu	0.93	0.44	<0.001
Horu	0.04	0.02	0.39
Hych	0.52	0.28	0.03
Mior	0.09	0.04	0.16
Peni	1	0.46	<0.001
Raca	0.68	0.24	<0.001
Razh	0.57	0.27	0.01
Syla	0.03	0.01	0.28

**Table 3:** Results of the Pearson regression for frog species diversity in the Zhoushan Archipelago as related to natural factors.

Influencing Factors	Pearson Regression			
	Log (Number of Species)		Number of Species	
	r	*p*	r	*p*
Log (area)	0.8718	<0.0001	0.898	<0.0001
Log (distance to mainland)	−0.3284	0.1462	−0.2901	0.2021
Log (distance to large island)	−0.0582	0.8021	−0.003	0.9895
Log (altitude)	0.5573	0.0087	0.5756	0.0063

## Data Availability

The data supporting the results are deposited in the Supplementary Materials.

## Supplementary Materials

The following supporting information can be downloaded at https://www.mdpi.com/article/10.3390/ani14111519/s1, Table S1: The related natural factors of Zhoushan archipelago. DTM = distance to mainland, DTLI = distance to large island; Table S2: The distribution of amphibians on Zhoushan Archipelago detected by each method, “Buga” = “Bufo gargarizans”, “Femu” = “Fejervarya multistriata”, “Horu” = “Hoplobatrachus rugulosus”, “Hych” = “Hyla chinensis”, “Mior” = “Microhyla ornata”, “Peni” = “Pelophylax nigromaculatus”, “Raca” = “Rana catesbeiana”, “Razh” = “Rana zhenhaiensis”, “Syla” = “Sylvirana latouchii”; Table S3: A total of 253 regressions for each model.
