# Supplementary material for: eDNA Metabarcoding Reveals the Species–Area Relationship of Amphibians on the Zhoushan Archipelago
Source: Animals (Basel). 2024 May 21;14(11):1519. doi: 10.3390/ani14111519 (PMC11171295; doi:10.3390/ani14111519)
Supplement: Supplementary file 1 [file animals-14-01519-s001.zip › animals-2982182-supplementary.pdf]

Table S1. The related natural factors of Zhoushan archipelago. DTM = distance to mainland, DTLI = distance to large island.

Site	Area	dtm	dtli	Height	Landform
Zhoushan	468.7	9	9	503	island
Daishan	100	37	12	175	island
Qushan	59.9	58.5	11.8	250	island
Liuheng	92.8	7	7	299	island
Jintang	76.4	3.6	3.6	455.9	island
Xiushan	23	26.8	2.3	207	island
Cezi	14.9	16.5	2.4	275	island
Sijiao	22.3	54.5	45.3	217.8	island
Meishan	21.9	0.5	0.5	148	island
Putuo	12.3	23.8	2.5	291	island
Xiaochangtu	10.8	37.6	2.8	299	island
Taohua	41	8.8	8.8	539	island
Xiazhi	16.7	13.2	2	207	island
Mayi	2.2	11.5	11.5	157	island
Jinpin	1.7	56	0.4	132	island
Shengshan	4.6	90	8.8	213	island
Huni	1.4	8.9	2.8	88	island
Fodu	7	7	2.3	183	island
Huanglong	5	68	3.3	223	island
Huaniao	3	75.5	15	236.9	island
Dongji	2.7	100	100	200	island
Chaiqiao	284	0	39.8	502.9	mainland
Guoju	359	0	36.5	309	mainland
Xiepu	195	0	84.7	69.9	mainland

Table S2. The distribution of amphibians on Zhoushan archipelago detected by each method; "**Buga**" = "**Bufo gargarizans**", "**Femu**" = "**Fejervarya multistriata**", "**Horu**" = "**Hoplobatrachus rugulosus**", "**Hych**" = "**Hyla chinensis**","**Mior**" = "**Microhyla ornata**", "**Peni**" = "**Pelophylax nigromaculatus**", "**Raca**" = "**Rana catesbeiana**", "**Razh**" = "**Rana zhenhaiensis**", "**Syla**" = "**Sylvirana latouchii**"

Site	Buga	Femu	Horu	Hych	Mior	Peni	Raca	Razh	Syla	Method
Cezi	1	1	0	1	1	1	1	1	0	eDNA
Chaiqiao	1	1	1	1	1	1	1	1	1	eDNA
Daishan	1	1	0	1	1	1	1	1	1	eDNA
Dongji	1	1	0	0	0	1	0	0	0	eDNA
Fodu	1	1	0	0	0	1	1	0	0	eDNA
Guoju	1	1	1	1	1	1	1	1	1	eDNA
Huanglong	1	1	0	0	0	1	0	0	0	eDNA
Huaniao	1	1	0	0	0	1	0	0	0	eDNA
Huni	1	1	0	0	0	1	0	0	0	eDNA
Jinpin	1	1	0	0	0	1	1	0	0	eDNA
Jintang	1	1	0	1	1	1	1	1	0	eDNA
Liuheng	1	1	0	1	1	1	1	1	0	eDNA
Mayi	1	1	0	0	0	1	1	0	0	eDNA
Meishan	1	1	0	1	0	1	1	0	0	eDNA
Putuo	1	1	0	1	0	1	1	1	0	eDNA
Qushan	1	1	0	1	1	1	1	1	0	eDNA
Shengshan	1	1	0	0	0	1	0	0	0	eDNA

Sijiao	1	1	0	1	0	1	1	1	0	eDNA
Taohua	1	1	0	1	1	1	1	1	0	eDNA
Xiaochangtu	1	1	0	0	0	1	1	1	0	eDNA
Xiazhi	1	1	0	1	0	1	1	1	0	eDNA
Xiepu	1	1	1	1	1	1	1	1	1	eDNA
Xiushan	1	1	0	1	1	1	1	1	0	eDNA
Zhoushan	1	1	0	1	1	1	1	1	1	eDNA
Cezi	1	1	0	1	0	1	1	1	0	TLTM
Chaiqiao	1	1	1	1	1	1	1	1	1	TLTM
Daishan	1	1	0	1	1	1	1	1	0	TLTM
Dongji	0	1	0	0	0	1	0	0	0	TLTM
Fodu	0	1	0	0	0	1	0	0	0	TLTM
Guoju	1	1	1	1	1	1	1	1	1	TLTM
Huanglong	1	1	0	0	0	1	0	0	0	TLTM
Huaniao	1	1	0	0	0	1	0	0	0	TLTM
Huni	0	1	0	0	0	1	0	0	0	TLTM
Jinpin	1	1	0	0	0	1	0	0	0	TLTM
Jintang	1	1	0	1	1	1	1	1	0	TLTM
Liuheng	1	1	0	1	1	1	1	1	0	TLTM
Mayi	1	1	0	0	0	1	0	0	0	TLTM
Meishan	1	1	0	1	0	1	0	0	0	TLTM
Putuo	0	1	0	1	0	1	1	1	0	TLTM
Qushan	1	1	0	1	1	1	0	1	0	TLTM
Shengshan	1	1	0	0	0	1	0	0	0	TLTM
Sijiao	1	1	0	0	0	1	1	1	0	TLTM
Taohua	1	1	0	1	0	1	1	0	0	TLTM
Xiaochangtu	1	1	0	0	0	1	0	1	0	TLTM
Xiazhi	1	1	0	1	0	1	1	0	0	TLTM
Xiepu	1	1	1	1	0	1	1	1	1	TLTM
Xiushan	1	1	0	1	1	1	1	1	0	TLTM
Zhoushan	1	1	0	1	1	1	1	1	0	TLTM

Table S3. 253 regressions for each model.

Model	T	R^2
1	0.146128036	0.796189087
2	0.156128036	0.79622799
3	0.166128036	0.796251071
4	0.176128036	0.796258223
5	0.186128036	0.796249341
6	0.196128036	0.79622432
7	0.206128036	0.796183054
8	0.216128036	0.796125441
9	0.226128036	0.79605138
10	0.236128036	0.796356794
11	0.246128036	0.796940571
12	0.256128036	0.797499323
13	0.266128036	0.798032666
14	0.276128036	0.798540215
15	0.286128036	0.799021585
16	0.296128036	0.799476393
17	0.306128036	0.799904254

18	0.316128036	0.800304785
19	0.326128036	0.800677604
20	0.336128036	0.801022329
21	0.346128036	0.801546307
22	0.356128036	0.802392239
23	0.366128036	0.803205627
24	0.376128036	0.803985744
25	0.386128036	0.804731858
26	0.396128036	0.80544323
27	0.406128036	0.806119121
28	0.416128036	0.806758786
29	0.426128036	0.807361476
30	0.436128036	0.807750517
31	0.446128036	0.80790353
32	0.456128036	0.80801136
33	0.466128036	0.808072957
34	0.476128036	0.808087261
35	0.486128036	0.807666448
36	0.496128036	0.807150195
37	0.506128036	0.806579652
38	0.516128036	0.805953471
39	0.526128036	0.805270289
40	0.536128036	0.804528727
41	0.546128036	0.803727391
42	0.556128036	0.802864872
43	0.566128036	0.801939749
44	0.576128036	0.800950588
45	0.586128036	0.799895944
46	0.596128036	0.798774361
47	0.606128036	0.797584376
48	0.616128036	0.796324516
49	0.626128036	0.794993301
50	0.636128036	0.793589248
51	0.646128036	0.792110868
52	0.656128036	0.79055667
53	0.666128036	0.7886909
54	0.676128036	0.786281037
55	0.686128036	0.783784002
56	0.696128036	0.78119802
57	0.706128036	0.777982322
58	0.716128036	0.774455957
59	0.726128036	0.770830242
60	0.736128036	0.767103157
61	0.746128036	0.76327268
62	0.756128036	0.759336783
63	0.766128036	0.755293441
64	0.776128036	0.75114063
65	0.786128036	0.746876335
66	0.796128036	0.742498552
67	0.806128036	0.738005293
68	0.816128036	0.733394588

69	0.826128036	0.728664491
70	0.836128036	0.723813085
71	0.846128036	0.718836184
72	0.856128036	0.71371398
73	0.866128036	0.708464266
74	0.876128036	0.703084776
75	0.886128036	0.697573272
76	0.896128036	0.691927547
77	0.906128036	0.686145438
78	0.916128036	0.680224825
79	0.926128036	0.674163648
80	0.936128036	0.667959906
81	0.946128036	0.661611675
82	0.956128036	0.655117108
83	0.966128036	0.648474454
84	0.976128036	0.64168206
85	0.986128036	0.634738388
86	0.996128036	0.627642023
87	1.006128036	0.620391687
88	1.016128036	0.612986249
89	1.026128036	0.605424741
90	1.036128036	0.597890816
91	1.046128036	0.59069566
92	1.056128036	0.583339561
93	1.066128036	0.575821087
94	1.076128036	0.56813898
95	1.086128036	0.560292175
96	1.096128036	0.553286194
97	1.106128036	0.54672866
98	1.116128036	0.540007591
99	1.126128036	0.533120864
100	1.136128036	0.526066515
101	1.146128036	0.518842756
102	1.156128036	0.511447994
103	1.166128036	0.503880857
104	1.176128036	0.496891455
105	1.186128036	0.491539754
106	1.196128036	0.486025922
107	1.206128036	0.480346901
108	1.216128036	0.474499749
109	1.226128036	0.468984382
110	1.236128036	0.464279915
111	1.246128036	0.459418652
112	1.256128036	0.454396814
113	1.266128036	0.449210682
114	1.276128036	0.443856619
115	1.286128036	0.43833109
116	1.296128036	0.432630688
117	1.306128036	0.426752156
118	1.316128036	0.42069242
119	1.326128036	0.414448616

120	1.336128036	0.408018123
121	1.346128036	0.401558227
122	1.356128036	0.39612115
123	1.366128036	0.391925433
124	1.376128036	0.389008432
125	1.386128036	0.385983151
126	1.396128036	0.382845383
127	1.406128036	0.379590788
128	1.416128036	0.376214892
129	1.426128036	0.372713086
130	1.436128036	0.369080635
131	1.446128036	0.36531268
132	1.456128036	0.361404243
133	1.466128036	0.357350239
134	1.476128036	0.353145485
135	1.486128036	0.348784712
136	1.496128036	0.344262583
137	1.506128036	0.339573708
138	1.516128036	0.334712671
139	1.526128036	0.329674049
140	1.536128036	0.32445245
141	1.546128036	0.319042543
142	1.556128036	0.313439096
143	1.566128036	0.307637026
144	1.576128036	0.301631448
145	1.586128036	0.295417733
146	1.596128036	0.288991571
147	1.606128036	0.282349047
148	1.616128036	0.276383431
149	1.626128036	0.272015569
150	1.636128036	0.267473456
151	1.646128036	0.262751266
152	1.656128036	0.257843236
153	1.666128036	0.252743716
154	1.676128036	0.247447225
155	1.686128036	0.241948522
156	1.696128036	0.236242673
157	1.706128036	0.230325142
158	1.716128036	0.224191886
159	1.726128036	0.217839458
160	1.736128036	0.211265124
161	1.746128036	0.204467001
162	1.756128036	0.197444189
163	1.766128036	0.19019693
164	1.776128036	0.182726773
165	1.786128036	0.177333341
166	1.796128036	0.172094148
167	1.806128036	0.166665756
168	1.816128036	0.161045992
169	1.826128036	0.155233489
170	1.836128036	0.149227824

171	1.846128036	0.14302966
172	1.856128036	0.136640908
173	1.866128036	0.130064895
174	1.876128036	0.123306538
175	1.886128036	0.117126727
176	1.896128036	0.112521997
177	1.906128036	0.107769148
178	1.916128036	0.102868567
179	1.926128036	0.097821555
180	1.936128036	0.092630449
181	1.946128036	0.087298755
182	1.956128036	0.081831286
183	1.966128036	0.076234304
184	1.976128036	0.072532922
185	1.986128036	0.069065317
186	1.996128036	0.065498989
187	2.006128036	0.064091681
188	2.016128036	0.064091681
189	2.026128036	0.064091681
190	2.036128036	0.064091681
191	2.046128036	0.064091681
192	2.056128036	0.064091681
193	2.066128036	0.064091681
194	2.076128036	0.064091681
195	2.086128036	0.064091681
196	2.096128036	0.064091681
197	2.106128036	0.064091681
198	2.116128036	0.064091681
199	2.126128036	0.064091681
200	2.136128036	0.064091681
201	2.146128036	0.064091681
202	2.156128036	0.064091681
203	2.166128036	0.064091681
204	2.176128036	0.064091681
205	2.186128036	0.064091681
206	2.196128036	0.064091681
207	2.206128036	0.064091681
208	2.216128036	0.064091681
209	2.226128036	0.064091681
210	2.236128036	0.064091681
211	2.246128036	0.064091681
212	2.256128036	0.064091681
213	2.266128036	0.064091681
214	2.276128036	0.064091681
215	2.286128036	0.064091681
216	2.296128036	0.064091681
217	2.306128036	0.064091681
218	2.316128036	0.064091681
219	2.326128036	0.064091681
220	2.336128036	0.064091681
221	2.346128036	0.064091681

222	2.356128036	0.064091681
223	2.366128036	0.064091681
224	2.376128036	0.064091681
225	2.386128036	0.064091681
226	2.396128036	0.064091681
227	2.406128036	0.064091681
228	2.416128036	0.064091681
229	2.426128036	0.064091681
230	2.436128036	0.064091681
231	2.446128036	0.064091681
232	2.456128036	0.064091681
233	2.466128036	0.064091681
234	2.476128036	0.064091681
235	2.486128036	0.064091681
236	2.496128036	0.064091681
237	2.506128036	0.064091681
238	2.516128036	0.064091681
239	2.526128036	0.064091681
240	2.536128036	0.064091681
241	2.546128036	0.064091681
242	2.556128036	0.064091681
243	2.566128036	0.064091681
244	2.576128036	0.064091681
245	2.586128036	0.064091681
246	2.596128036	0.064091681
247	2.606128036	0.064091681
248	2.616128036	0.064091681
249	2.626128036	0.064091681
250	2.636128036	0.064091681
251	2.646128036	0.064091681
252	2.656128036	0.064091681
253	2.666128036	0.064091681
